# Job satisfaction and associated factors among nurses in Bahir Dar city administrative, North West Ethiopia, 2017

**DOI:** 10.1186/s13104-019-4363-4

**Published:** 2019-06-07

**Authors:** Emiru Ayalew, Yinager Workineh

**Affiliations:** 1 Department of Adult Health Nursing, College of Medicine and Health Science, Bahir Dar University, Bahir Dar, Ethiopia; 2 Department of Child Health Nursing, College of Medicine and Health Science, Bahir Dar University, Bahir Dar, Ethiopia

**Keywords:** Job satisfaction, Hygienic, Motivational factors, Nurses

## Abstract

**Objective:**

To assess the level of job satisfaction and associated factors among nurses in Bahir Dar city, Northwest Ethiopia, 2017.

**Results:**

The overall proportion of nurses’ job satisfaction was 43.6%. From motivational factors, advancement (AOR = 2.64; 95% CI [1.17, 5.96]) and recognition (AOR = 2.56; 95% CI [1.08, 6.08]) were the main determinants of nurses’ job satisfaction. Among hygienic factors, work security (AOR = 4.88; 95% CI [1.13, 21.03]) was positively associated with nurses’ job satisfaction. In conclusion, the nurses’ job satisfaction was low in this study setting. Modifiable factors such as advancement, recognition and work security positively affect job satisfaction of nurses. Therefore, the current study recommended that the health care system administers should work on improvement of advancement, security, and recognition in the facilities.

## Introduction

Job satisfaction is a measure of cognitive and behavioral components of workers’ comfort with their job [1, 2]. It also refers to the outlook and spirits of professionals towards their work [3].

Physiological and psychological needs are the main factors of job satisfaction and dissatisfaction. Payment, supervision, fringe benefits, operating procedure, coworker and communication are physiological factors of job satisfaction. Similarly, the nature of work, promotion, and contingent are psychological factors. In addition to the above, work environment and supervision are hygienic factors of nurses’ job satisfaction [4]. In general, the determinants of job satisfaction of professionals are classified as intrinsic and extrinsic factors [5].

Job dissatisfaction affects the quality and cost of patient care due to nurses’ preservation from their tasks [6]. The dissatisfaction was higher in the hospital than other health facilities as evidenced by one in five nurses plan to depart their profession within a year [7].

The study conducted in Nigeria revealed that 67.1% of nurses are dissatisfied with their job [8]. A similar study done in Kampala, Uganda showed that 17.4% of nurses are satisfied [9]. Another study conducted in Malawi, Tanzania and South Africa stated 71%, 82.3%, and 52.1% of nurses are satisfied respectively [10].

Different studies were conducted to investigate the determinants of nurses’ job satisfaction. On this dimension, the study conducted in New Delhi showed that inadequate salary, lack of promotions, fringe benefits, training and rewards, poor working conditions, nature of work and coworkers were the main determinants of nurses’ job satisfaction [11]. Another similar study conducted in Ethiopia indicated that hospital nurses are dissatisfied [6, 11].

A shortage of experienced nurses reduced the quality of care in the health facilities [12]. This critical and global problem of the health care system will be solved by augmenting the job satisfaction of nurses at the organization as well as individual levels.

Even though nurses’ job satisfaction is the base for quality health care, factors of dissatisfaction and leaving of their job weren’t well investigated in Ethiopia before. Hence, the current study might add evidence for policymakers, health care administers and nurse managers to alleviate the barriers of job satisfaction in Ethiopia. Therefore, this study aimed to assess the level of nurses’ job satisfaction and associated factors in Bahir Dar city, Northwest Ethiopia, 2017.

## Main text

### Methods

#### Study design, setting and period

The cross-sectional study design was conducted in Bahir Dar city public health facilities from 1 to 30 March 2017. Bahir Dar (capital city of Amhara region) is 565 km apart from Addis Ababa. In the city, there were two hospitals and ten health centers with a total of 441 nurses during the study.

#### Sample size determination and procedure

The sample size was determined by using a single population proportion formula. The following assumptions, such as a 95% confidence interval (CI), 5% the margin of error (d), 10% non-response rate and 52.5% of proportion (p) were considered [13].

$${\text{N}} = \frac{{\left( {{\text{z }}\alpha / 2} \right)^2\times{\text{p}}\left( {1 - {\text{p}}} \right)}}{{{\text{d}}^{2} }}$$

$${\text{N}} = \frac{{\left( { 1. 9 6} \right) ^2\times 0. 5 2 5\left( { 1- 0. 5 2 5} \right)}}{{\left( {0.0 5} \right)^{ 2} }} = 3 8 3. 1 9 9 6\sim 3 8 3.$$

Finally, by using the correction formula and 10% non-response rate, the sample size was 226.

Two hospitals and ten health centers were selected from Bahir Dar city. Lists of nurses were obtained from each facility payroll. The sample sizes were proportionally allocated in each health institution based on the number of nurses. Then, the study participants were selected by simple random sampling methods. The nurses in annual, learning and maternity leave were excluded from the study.

#### Measurement tool

A structured self-administered questionnaire was used to collect the data. The authors measured the outcome variables based on Job satisfaction scale and Minnesota Questionnaire [12, 14]. Negative items were reversed to positive before summing. The measuring items on nurses’ job satisfaction were achievement, advancement, work itself, recognition, growth at work, organization policy, relationship with colleague and supervisor, payment and working condition. We calculated the scores of each factor based on the answers to all items. Finally, nurses’ job satisfaction was determined by using the demarcation threshold formula [15].

#### Data processing and analysis

We coded, entered, cleaned and analyzed the data using SPSS version 21. The frequency of each variable was calculated. The logistic regression model was used to determine statistical significance of variables. In this model, the odds ratio with 95% confidence interval was used to determine the strength of association between dependent and independent variables. The variables with a p-value of less or equal to 0.2 in bivariate analysis model were transferred to multivariate. Finally, we considered the p-values less than 0.05 as statistically significant.

### Results

#### Demographic profiles of respondents

Among 226 sampled nurses, 220 of them have participated with the response rate of 98.3%. In the current study, 106 (48.2%) and 133 (60.5%) nurses were females and married respectively. The mean age of participants was 30.05 (SD = ± 6.172) years. Regarding educational status, 121 (55%) nurses were BSc holders. Fifty-six (25.5%) nurses had ten or more years of work experience (Table 1).

**Table 1:** Socio-demographic characteristics of study participant at Bahir Dar, North west Ethiopia, June 2017

Variable	Category	Frequency	Percent
Sex	Male	114	51.8
	Female	106	48.2
Current age in year	20–30	142	64.5
	31–40	64	29.1
	41–50	13	5.9
	≥ 51	1	0.5
Marital status	Single	59	26.8
	Married	133	60.5
	Divorced	15	6.8
	Widowed	13	5.9
Religion	Orthodox Tewahido	126	57.3
	Protestant	37	16.8
	Muslim	28	12.7
	Other	29	13.2
Ethnicity	Amhara	189	85.9
	Oromo	14	6.4
	Other	17	7.7
Educational status	Diploma nurse	95	43.2
	BSc. nurse	121	55
	MSc.	4	1.8
Work experience	6 month to < 1 year	53	24.1
	1 year to < 2 years	29	13.2
	2 years to < 5 years	35	15.9
	5 years to < 10 years	47	21.4
	≥ 10 years	56	25.5
Institution	Hospital	138	62.3
	Health center	62	37.7

#### Nurses agreement with motivational and hygienic factors of job satisfaction

In this study, 27.7% of nurses reported that payment is hygienic factors of job satisfaction. Similarly, 79.5% of nurses stated that relationship with friends as hygienic factors. On the other hand, 69.1% and 68.6% of nurses disagreed on working conditions and work security respectively as hygienic factors of job satisfaction. In the current study, the overall job satisfaction was 43.6% (Table 2).

**Table 2:** Frequency distribution of nurses’ agreement with motivational and hygienic factors in Bahir Dar administrative city, 2017

Variables	Level of agreement in percent	
	Agreement (%)	Disagreement (%)
Achievement	64.5	34.5
Advancement	59.5	40.5
Work it self	75	25
Recognition	55.5	44.5
Growth at work	70	30
Organization policy	49.1	50.9
Work security	32.4	68.6
Relationship with friends	79.5	20.5
Relationship with supervisors	42.3	57.7
Payment	27.7	72.3
Working condition	30.9	69.1

#### Factors associated with job satisfaction

In this study, multivariate logistic regression analysis indicated that advancement, work security and recognition were the main determinants of nurses’ job satisfaction. From all motivating factors, advancement (AOR = 2.64; 95% CI [1.17, 5.96]) and recognition (AOR = 2.56; 95% CI [1.08, 6.08]) were positively associated with job satisfaction of nurses as compared with its counterparts. Among hygiene factors, work security (AOR = 4.88; 95% CI [1.13, 21.03]) was the only determinants of nurses’ job satisfaction (Table 3).

**Table 3:** Results from Bivariate and multiple logistic regression analysis about nursing satisfaction in Bahir Dar administrative city, June 2016

Variables	Categories	Level of satisfaction		Odds ratio (95% confidence interval)	
		Satisfied	Not satisfied	COR (95% CI)	AOR (95% CI)
		N (%)	N (%)		
Work experience	6 month–1 year	19 (35.8)	34 (64.2)	1.00	1.00
	1–2 year	10 (34.5)	19 (65.5)	0.94 (0.36, 2.43)	0.82 (0.23, 2.96)
	2–5 year	17 (51.4)	18 (48.6)	1.69 (0.71, 4.03)	0.68 (0.19, 2.43)
	5–10 year	26 (55.3)	21 (44.7)	2.216 (0.99, 4.95)	1.51 (0.49, 4.63)
	≥ 10 year	24 (42.9)	32 (57.1)	1.342 (0.620, 2.903)	0.73 (0.24, 2.22)
Achievement	Agree	81 (57)	61 (43)	5.56 (2.90, 10.73)*	2.21 (0.86, 5.64)
	Disagree	15 (19.2)	63 (80.8)	1.00	1.00
Advancement	Agree	61 (68.5)	28 (31.5)	5.98 (3.31, 10.79)*	2.64 (1.17, 5.96)*
	Disagree	35 (26.7)	96 (73.3)	1.00	1.00
Work itself	Agree	87 (52.7)	78 (47.3)	5.70 (2.62, 12.39)*	1.60 (0.55, 4.66)
	Disagree	9 (16.4)	46 (83.6)	1.00	1.00
Growth at work	Agree	82 (53.2)	72 (46.4)	4.23 (2.17, 8.26)*	1.63 (0.65, 4.09)
	Disagree	14 (21.2)	52 (78.8)	1.00	1.00
Recognition at work	Agree	78 (63.9)	44 (36.1)	7.88 (4.19, 14.81)*	2.56 (1.08, 6.08)*
	Disagree	18 (18.4)	80 (80.6)	1.00	1.00
Organization policy	Agree	71 (65.7)	37 (34.3)	6.68 (3.68, 12.13)*	1.63 (0.64, 4.14)
	Disagree	25 (22.3)	87 (77.7)	1.00	1.00
Relationship with friends	Agree	88 (50.3)	87 (49.3)	4.68 (2.06, 10.62)*	0.99 (0.32, 3.09)
	Disagree	8 (17.8)	37 (82.2)	1.00	1.00
Work security	Agree	60 (87)	9 (13)	21.26 (9.62, 47.13)*	4.88 (1.13, 21.03)*
	Disagree	36 (23.8)	115 (76.2)	1.00	1.00
Payment	Agree	51 (83.6)	10 (16.4)	12.92 (6.04, 27.64)*	2.64 (0.73, 9.62)
	Disagree	45 (28.3)	114 (71.7)	1.00	1.00
Relationship with supervisor	Agree	61 (65.6)	32 (34.4)	5.01 (2.81, 8.94)*	1.85 (0.82, 4.17)
	Disagree	35 (27.6)	92 (72.4)	1.00	1.00
Working condition	Agree	56 (82.4)	12 (17.6)	13.07 (6.36, 26.86)*	0.63 (0.14, 2.85)
	Disagree	40 (26.3)	112 (73.7)	1.00	1.00

Italicized—significantly associated value

*Statistically significant p < 0.05

### Discussion

The determination of job satisfaction and associated factors among nurses in Bahir Dar, North West Ethiopia was the main objective of the current study. Based on this aim, the job satisfaction of nurses was 43.6%. This finding is similar to studies done in Turkey, Pakistan, and Ethiopia [15–17]. On the other corner, it is higher than studies conducted in Nigeria (32.9%) and Uganda (17.4%) [8, 9], but lower than the study done in, Malawi (71.0%), Tanzania (82.6%), South Africa (52.1%) and Sidama (52.5%) [10, 13]. The possible reasons for this difference might be due to variation in the study setting, educational level, measuring tools and salary between the current and previous studies.

In this study, the determinants of nurses’ job satisfaction were assessed. The result pointed out that the advancement was the positive risk factors of nurses’ job satisfaction. This is in line with the study done in England [5]. The high level of job satisfaction in advancement might be due to the nurses’ perception of gradual improvement or development.

Similarly, the current study revealed that recognition as a motivational factor had a positive association with job satisfaction of nurses. The finding agrees with studies conducted in England, India, Pakistan, and Ethiopia [5, 15, 16, 18]. Hence, constructive recognition should be given to promote job satisfaction of employees.

Work security was the last but not the least factor of nurses’ job satisfaction. In this case, work security positively promoted the satisfaction of nurses. This is in agreement with the studies carried out in China, Turkey, and Ethiopia [1, 17, 19]. The high level of job satisfaction in work security might be due to its positive effect on physical and psychological comforts of nurses.

### Conclusion

The nurses’ job satisfaction was low in this study setting. Modifiable factors such as advancement, recognition and work security increased job satisfaction of nurses. Therefore, the current study recommended that the health care system administers should work on improvement of advancement, security, and recognition in the facilities.

## Limitations

Since this study assessed motivation and satisfaction related issues, it might bring emotional responses. Application of cross-sectional study could not detect the cause-effect relationship between the variables.

## Data Availability

The data of this study can’t be shared publicly due to presence of sensitive (confidential) participants’ information.

## Abbreviations

AOR: adjusted odds ratio
CI: confidence interval
COR: crowd odd ratio
SPSS: statistical

## References

[1] Onuoha P, Stephen A, Bernard P, Corban A, Mahabir M, Israel-Richardson D (2017). Factors that contribute to work motivation and job satisfaction among hospital nurses in Trinidad and Tobago. Int J Health Sci Res..

[2] Hulin CL, Judge TA (2003). Job attitudes. Handbook of psychology: industrial and organizational psychology.

[3] Aziri B (2011). Job satisfaction: a literature review. Manag Res Pract.

[4] Rosales RA, Labrague LJ, Rosales GL (2013). Nurses’ job satisfaction and Burnout: is there a connection?. Int J Adv Nurs Stud.

[5] Lephalala R, Ehlers VJ, Oosthuizen MJ (2008). Factors influencing nurses’ job satisfaction in selected private hospitals in England. Curationis.

[6] Gulavani A, Shinde M (2014). Occupational stress and job satisfaction among nurses. Int J Sci Res (IJSR).

[7] Berhe H (2014). Job satisfaction of nurses and associated factors in public hospitals in Tigray Region, Northern Ethiopia. Greener J Med Sci.

[8] Ayamolowo SJ (2013). Job satisfaction and work environment of primary health care nurses in Ekiti State, Nigeria: an exploratory study. Int J Caring Sci.

[9] Nabirye RC, Brown KC, Pryor ER, Maples EH (2011). Occupational stress, job satisfaction and job performance among hospital nurses in Kampala, Uganda. J Nurs Manag.

[10] Blaauw D, Ditlopo P, Maseko F, Chirwa M, Mwisongo A, Bidwell P, Thomas S, Normand C (2013). Comparing the job satisfaction and intention to leave of different categories of health workers in Tanzania, Malawi, and South Africa. Glob Health Action.

[11] Kohli S, Bagga R (2013). Job satisfaction amongst contractual and regular nursing staff in two government hospitals of Delhi: a comparison. Health Popul Perspect Issues.

[12] Baylor KM. The influence of intrinsic and extrinsic job satisfaction factors and affective commitment on the intention to quit for occupations characterized by high voluntary attrition; 2010.

[13] Asegid A, Belachew T, Yimam E (2014). Factors influencing job satisfaction and anticipated turnover among nurses in Sidama zone public health facilities, South Ethiopia. Nurs Res Pract.

[14] Weiss DJ, Dawis RV, England GW (1967). Manual for the Minnesota satisfaction questionnaire. Minnesota studies in vocational rehabilitation.

[15] Yami A, Hamza L, Hassen A, Jira C, Sudhakar M (2011). Job satisfaction and its determinants among health workers in Jimma university specialized hospital, southwest Ethiopia. Ethiop J Health Sci.

[16] Kumar R, Ahmed J, Shaikh BT, Hafeez R, Hafeez A (2013). Job satisfaction among public health professionals working in public sector: a cross sectional study from Pakistan. Hum Resour Health.

[17] Masum AKM, Azad MAK, Hoque KE, Beh L-S, Wanke P, Arslan Ö (2016). Job satisfaction and intention to quit: an empirical analysis of nurses in Turkey. PeerJ.

[18] Ali N, Ali A (2014). The mediating effect of job satisfaction between psychological capital and job burnout of Pakistani nurses. Pak J Commer Soc Sci.

[19] Bekru ET, Cherie A, Anjulo AA (2017). Job satisfaction and determinant factors among midwives working at health facilities in Addis Ababa city, Ethiopia. PLoS ONE.

